# Clinical validation of artificial intelligence algorithms for the detection of different central-involved retinal pathologies and glaucoma from non-mydriatic images

**DOI:** 10.3389/frai.2026.1754682

**Published:** 2026-03-10

**Authors:** Josep Vidal-Alaball, Alba Arocas Bonache, Jordi Solé-Casals, Didac Royo Fibla, Francesc Xavier Marin-Gomez, Laura Natalia Distéfano, Anna Boixadera, Ángela Casado-García, Manuel García-Domínguez, Adrián Inés, Jonathan Heras, Miguel Angel Zapata

**Affiliations:** 1 Innovation and Research Unit, Heath Catalan Institute, Manresa, Spain; 2 Intelligence for Primary Care Research Group, The Foundation University Institute for Primary Health Care Research Jordi Gol i Gurina, Manresa, Spain; 3 Department of Medicine, Faculty of Medicine, University of Vic - Central University of Catalonia, Vic, Spain; 4 Primary Care Center SantPedor, Heath Catalan Institute, Manresa, Spain; 5 Data and Signal Processing Group, Faculty of Science, Technology and Engineering, University of Vic - Central University of Catalonia, Vic, Spain; 6 Department of Psychiatry, University of Cambridge, Cambridge, United Kingdom; 7 UPRetina, Barcelona, Spain; 8 Primary Care Center Osona, Heath Catalan Institute, Vic, Spain; 9 Ophthalmology Department, University Hospital Vall d'Hebron, Barcelona, Spain; 10 Department of Mathematics and Computer Science, University of La Rioja, Logroño, Spain

**Keywords:** artificial intelligence, clinical validation, computer vision, retinal diseases, retinal fundus image

## Abstract

The use of Artificial intelligence (AI) algorithms for detecting different ophthalmic diseases, especially diabetic retinopathy (DR), has become increasingly popular. In this paper, we evaluate the screening performance of different AI algorithms based on convolutional neural networks (CNNs) in a real-world scenario. To that aim, we conducted an observational and cross-sectional study on patients aged ≥18 years with type-2 diabetes mellitus, who had undergone fundus examination for DR screening using a teleophthalmology program. We used the UPRETINA diagnostic system, which consists of 8 AI algorithms based on CNNs. A total of 1,652 eyes from 871 patients were analyzed. The AI algorithms had a sensitivity/specificity of 86.8%/95.6% for detecting DR; 94.9%/94.3% for detecting age-related macular degeneration (AMD); 82.7%/92.4% for detecting glaucomatous optic neuropathy (GON); 87.0%/87.5% for detecting epiretinal membrane; and 89.7%/98.0% for detecting nevus. Additionally, the sensitivity/specificity for correctly classifying images as right eye/left eye and to correctly classifying images gradeability (medium or high quality) were 100% /100 and 92.9%/90.5%, respectively. The AUROC of the AI algorithms ranged between 0.9777 (AMD) and 0.9122 (GON). UPRETINA system was capable of automatically and accurately classifying the screening retinographies, reducing workload and leading to a scenario of more efficient optimization of resources.

**Clinical trial registration:**

https://clinicaltrials.gov/study/NCT04132401 NCT04132401.

## 1 Introduction

The aging of the population is associated with an increase in the prevalence of age-related diseases. By 2030, one in six people in the world will be aged 60 years or over (World Health Organization, 2025).

Prevalence of diabetes mellitus (DM) and, therefore, chronic, and acute DM related complications, has significantly increased all over the world (Ogurtsova et al., 2017; Simó-Servat et al., 2019; Harding et al., 2019). Additionally, prevalence of other age-related retinal diseases, like, for example, age-related macular degeneration (AMD) has increased over the last decades (Mitchell et al., 2018). In fact, the number of European Union inhabitants with any AMD is expected to increase from 67 to 77 million by 2050; while the incidence of late AMD is estimated to increase from 400,000 per year today to 700,000 per year in 2050 (Li et al., 2020).

Because many of these pathologies appear and evolve asymptomatically for years, the importance of early diagnosis and appropriate treatment of ophthalmic diseases has never been greater (Simó-Servat et al., 2019; Harding et al., 2019; Mitchell et al., 2018).

Diabetic retinopathy (DR) is a common sight threatening complication of DM which remains a leading cause of visual loss in working-age populations (Simó-Servat et al., 2019; Mitchell et al., 2018). The first approach to DR diagnosis is based on its clinical manifestations of retinal vascular damage.

The primary method for diagnosing and assessing DR involves direct and indirect ophthalmoscopy. Among the different imaging techniques used currently, color fundus photography has been identified as a useful tool for screening purposes in DR and AMD (Cavallerano et al., 2005; Vujosevic et al., 2009; Zapata et al., 2021). Moreover, the development of newer techniques, such as stereoscopic imaging, nonmydriatic camera, and mobile phone-based fundus camera have emerged as valuable tools for diagnosing and/or monitoring the clinical course of DR (Tennant et al., 2001; Liesenfeld et al., 2000).

The development of different diagnostic tools, such as optical coherence tomography (OCT) and non-mydriatic fundus camera, has made significant advances in the management of these diseases (Zapata et al., 2021; Baskin, 2010; Liu et al., 2014; Chan et al., 2015). However, the interpretation and management of retinal diseases have largely become more complex for ophthalmologists, by virtue of the large accumulation of images and findings leading to a “big data” challenge (Obermeyer and Lee, 2017; Pellegrini et al., 2020; Ferro Desideri et al., 2022).

It is noteworthy that approximately 30% of patients with DM had never undergone a fundus examination (Cavallerano et al., 2005; Vujosevic et al., 2009; Garg and Davis, 2009).

Over the last several years significant advances in telecommunications, artificial intelligence (AI), and machine learning (ML)-based systems have opened new directions for creating efficient tools to evaluate different ophthalmic diseases, which may help to optimize health-care resources (Ferro Desideri et al., 2022; Schmidt-Erfurth et al., 2018; Dankwa-Mullan et al., 2019). Moreover, there is increasing evidence suggesting that AI may achieve similar, or even greater, performance for detecting DR (Somfai et al., 2014; Raman et al., 2019), classifying AMD (Venhuizen et al., 2017), or diagnosing glaucoma (Ittoop et al., 2022).

While there has been a proliferation of studies examining the application of AI in screening for ocular diseases such as diabetic retinopathy, glaucoma, and other center-involved retinal diseases, the majority of these have been conducted in controlled settings or with retrospective data. As far as we know, only few AI algorithms have received validation by the Food and Drug Administration (FDA) (Zarranz-Ventura, 2020) for such purposes and there remains a significant gap in the literature regarding clinical studies that rigorously validate algorithms in real clinical practice (Domalpally and Channa, 2021).

Because data about the use of AI for screening of DR, glaucoma, and other center-involved retinal diseases in clinical-practice are limited, we wanted to evaluate the UPRETINA diagnostic system, which consists of 8 AI algorithms based on convolutional neural networks (CNN’s) (Zarranz-Ventura, 2020; Domalpally and Channa, 2021; Zapata et al., 2020; Font et al., 2022; Casado-García et al., 2021).

The novelty of this manuscript does not lie in proposing new neural network architectures, but rather in the real-world clinical validation of an AI-based screening system (UPRETINA) integrated into a primary care teleophthalmology program. Specifically: (i) we evaluate the diagnostic performance of a multi-pathology system in consecutive type 2 diabetes patients across three primary care centers in Catalonia (May–August 2021); (ii) we compare the system performance against primary care physicians readings and against the reference standard (gold standard) defined by retina specialists; (iii) we evaluate functional workflow modules (e.g., quality/gradeability and laterality), in addition to pathology detection; and (iv) we report diagnostic accuracy metrics (AUROC, sensitivity/specificity with 95% CI, kappa agreement, and F1-score), as well as the image inclusion/exclusion flow.

## 2 Methods

### 2.1 Study design

Observational and cross-sectional study conducted on consecutive patients with type-2 DM, who had undergone fundus examination for DR screening using a teleophthalmology program, between May and August 2021 in three primary care centers of Catalonia. We followed the STROBE Guidelines.

This study constitutes a real-world clinical validation of the UPRETINA AI system. The validation protocol employed triple reading (AI system, primary care physicians, and reference standard by retina specialists with discrepancy resolution), enabling direct comparison of diagnostic performance across all evaluated pathologies.

The study was conducted in accordance with the rules of the Declaration of Helsinki and all the study participants provided written informed consent before starting the study. The University Institute for Research in Primary Health Care Jordi Gol i Gurina (Barcelona, Spain) ethics committee approved the trial study protocol (approval code: P18/109). This study was registered in ClinicalTRials. Gov with the number NCT04132401.

Any information that could lead to an individual being identified has been encrypted or removed, as appropriate, to guarantee their anonymity.

Detailed information on the protocol of this study has been published elsewhere (Vidal-Alaball et al., 2019).

### 2.2 Sample size

Our study involved a comprehensive analysis of 902 patients. Notably, since DR was the main lesion to be excluded in our patients, we focused our sample size calculation on this pathology; we mirrored the sample size used in the study by Abràmoff et al. (2018) which investigated AI-based diagnostics in primary care, providing a reliable precedent. The sample size was formally estimated based on expected diagnostic performance for DR. Using previously reported values of sensitivity (~0.85–0.90), specificity (~0.95), and the known prevalence of DR in type 2 diabetes screening populations (approximately 12–20%), a precision-based calculation indicated that slightly more than 1,000 screened cases would be required to estimate sensitivity with a margin of error of ±5% at the 95% confidence level. The final study cohort (*n* = 1,652 eyes) exceeded this requirement, supporting adequate precision of the DR performance estimates.

### 2.3 Inclusion/exclusion criteria

This study included patients aged ≥18 years with clinical diagnosed type-2 DM, who were attended for DR screening, in three Catalonian primary care centers.

Patients with routine ophthalmological reviews due to other causes; patients with neurological, physical and/or psychiatric disorders who were unable to comply with the protocol indications; patients with systemic and/or ophthalmic diseases that interfere with the image’s quality, were excluded from the study.

### 2.4 Acquisition protocol

Color fundus photos were acquired to the UPRETINA platform.

Two fundus images per patient (one image per eye), centered between macula and optic disc, were taken with a non-mydriatic camera (Topcon TRC-NW400; Topcon SA, Barcelona, Spain). 80% of the cases were performed without pupillary dilatation, while the remaining 20% (all performed in the same primary care center) were dilated with a drop of tropicamide (Vidal-Alaball et al., 2019).

A triple labeling of the fundus images has been carried out; a reading using the UPRETINA algorithm, another reading performed by the primary-care specialists, and finally another reading, which was considered as “gold standard,” performed by retina specialist ophthalmologists (Vidal-Alaball et al., 2019).

### 2.5 Human grading and screening protocol

**Figure 1:**
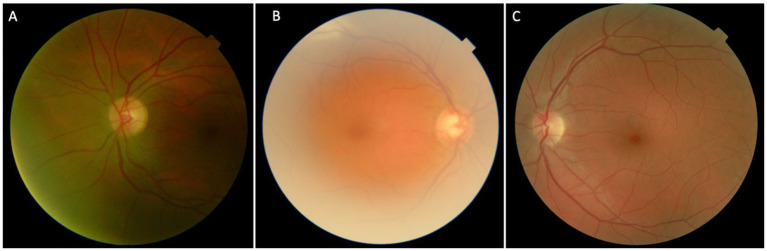
Different categories in which the images were defined: **(A)** Noise/low-quality; **(B)** Medium quality; **(C)** High quality.

The initial screening for image quality was performed using artificial intelligence-based software developed and described in our previous work (Zapata et al., 2020; Vidal-Alaball et al., 2019). This software evaluates the quality of images based on predefined criteria that focus on the clarity and diagnostic utility of the image. The images were classified according to their quality into three categories. (A) Noise/Low quality images were those with significant artifacts or blurriness precluding any retinal assessment and thus were not evaluated; (B) Medium quality images permitted retinal structure identification despite minor imperfections and required careful consideration during grading. (C) High-quality images provided clear visibility of retinal details without significant noise or artifacts, allowing for confident assessment (Figure 1).

At the reading center, retinal images were graded by two independent retinal specialists, with more than 10 years of experience and who have passed a certification for the evaluation of retinal images. If there was no agreement between them, the image was evaluated by a third retina specialist (who also has experience ≥10 years and has passed a certification for the evaluation of retinal images) to make the final determination.

The evaluators only made one determination of each image. The current study did not assess Retina specialists intra observer reliability. Nevertheless, their degree of intra-observer reliability was greater than 79% (Font et al., 2022).

Additionally, general practitioners performed a single evaluation of the images, without the possibility of evaluating patients again. The general practitioners participating in the study USUALLY perform fundus image readings of their patients, following the diabetic screening protocols of the Institut Catala de la Salut. Although primary care physicians in this program are primarily trained for diabetic retinopathy detection, they are also instructed to identify other relevant retinal and optic nerve abnormalities, and the screening software includes specific fields to report suspected AMD, nevus, and glaucomatous optic nerve findings. All comparisons were performed against a full ophthalmological examination, which served as the gold standard.

**Figure 2:**
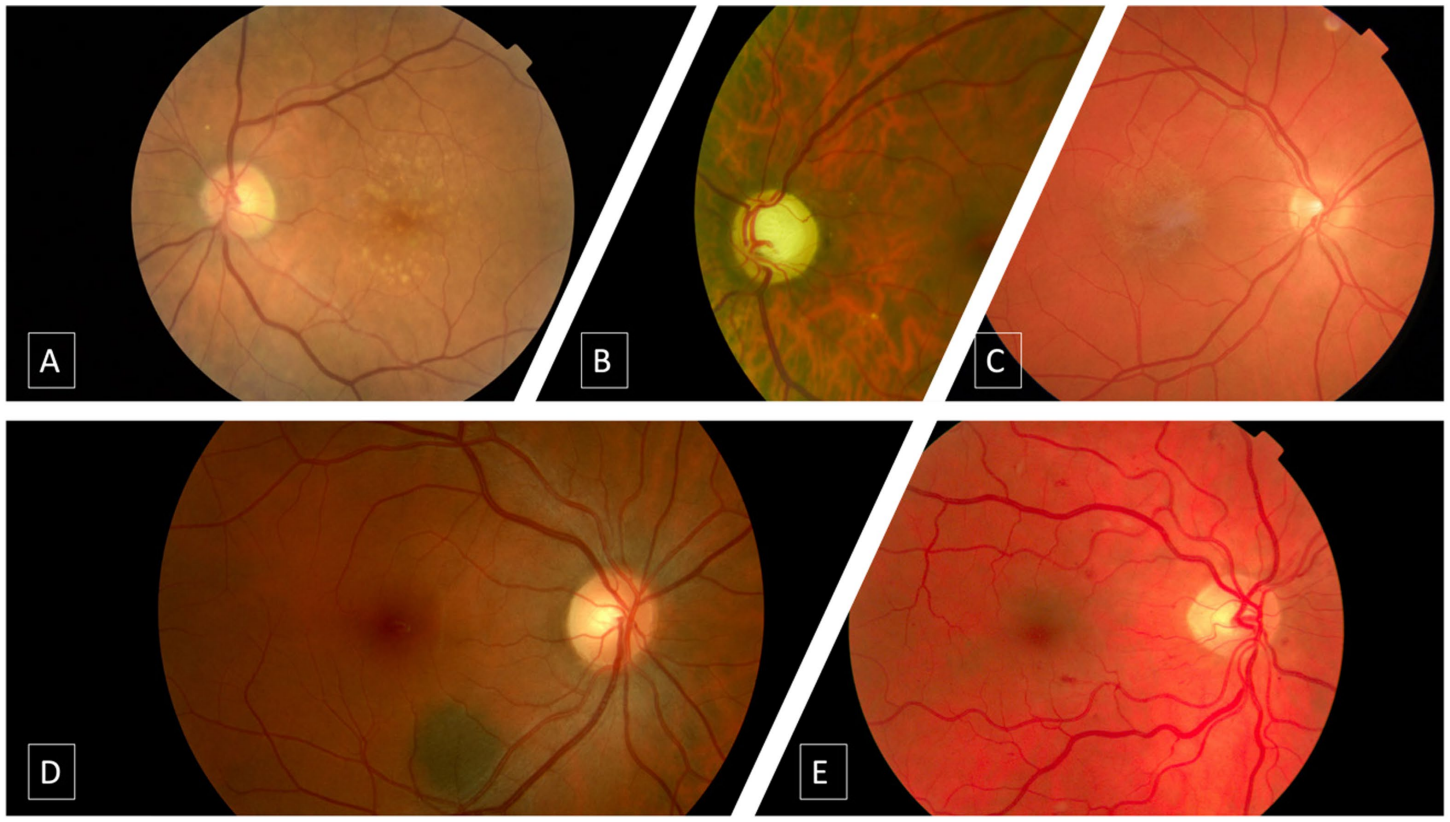
Typical imaging characteristics of studied pathologies. **(A)** Intermediate age-related macular degeneration. **(B)** Glaucomatous optic neuropathy. **(C)** Epiretinal membrane. **(D)** Choroidal nevus. **(E)** Moderate non proliferative diabetic retinopathy.

DR and DME were graded according to the international clinical diabetic retinopathy scale (Wilkinson et al., 2003), which classifies diabetic retinopathy according to the severity of microaneurysms and hemorrhages in 5 steps: no diabetic retinopathy (no abnormalities), mild NPDR, moderate NPDR, severe NPDR, and PDR. AMD, GON, nevus, and epiretinal membrane were classified in a binary manner based on the presence or absence of signs; the specifications are described in previous publications by Zapata et al. (2020), Font et al. (2022). To define the presence of macular degeneration, the international classification published in 2013 was followed, considering any type of AMD; early, intermediate, or advanced. The binary algorithm detects any type of AMD (early, intermediate or advanced). The GON algorithm detected a suspicious glaucoma optic disc, considered by a cup-to-disc ratio of 0.7 or more in the vertical axis and/or other typical changes caused by glaucoma, such as localized notches or RNFL defects. Epiretinal membranes were determined by the presence of folds and macular sheen, and the nevus algorithm, also binary, was determined by the presence of round or oblong lesions, deep to the retina and retinal pigment epithelium (RPE), with a grayish or brownish appearance, flat or slightly elevated, with or without drusen. Figure 2 presents representative images for all studied diseases.

### 2.6 Automated grading

The UPRETINA diagnostic system was used (Zapata et al., 2020; Font et al., 2022; Casado-García et al., 2021; Vidal-Alaball et al., 2019). This system consists of 8 AI algorithms based on CNN’s. The screening algorithm has 5 independently trained neural networks that target specific ophthalmic pathologies (AMD, DR, glaucoma optic neuropathy, epiretinal membrane, and Nevus). Additionally, a sixth algorithm, containing images of the aforementioned ophthalmic pathologies and other undetermined maculopathies, works as an outlier detector (Zapata et al., 2020; Font et al., 2022). The different neural networks evaluate each image and emit a combined response. If the algorithm detects any pathology, the examined image is classified as Abnormal.

System pipeline description. The UPRETINA inference pipeline processes each fundus image through a series of modules. First, images undergo quality assessment (classified as Noise/Low, Medium, or High). Noise/Low quality images are classified as non-gradable and excluded from analysis. Gradable images (Medium/High quality) proceed through laterality detection (OD/OS), followed by parallel pathology detection using five specialized models (DR, AMD, GON, ERM, Nevus). An outlier detector identifies out-of-distribution cases. The response combination module integrates all outputs to produce the final classification (Normal/Abnormal), pathology labels, and referral recommendation.

**Figure 3:**
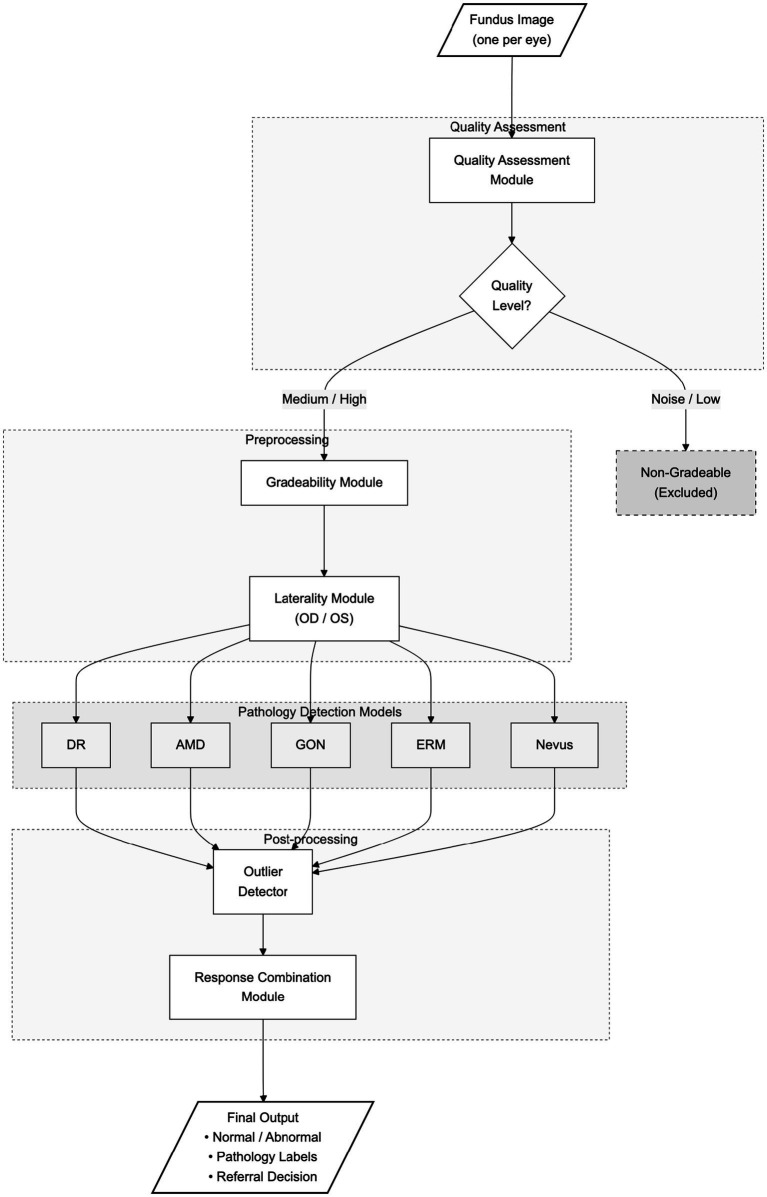
UPRETINA system architecture and inference pipeline. The diagram illustrates the complete flow from fundus image acquisition to final clinical output. Images are first assessed for quality (Noise/Low, Medium, High). Noise/Low quality images are classified as non-gradable and excluded from analysis. Gradable images proceed through laterality detection (OD/OS), followed by parallel pathology detection using five specialized models (DR, AMD, GON, ERM, Nevus). An outlier detector identifies out-of-distribution cases. The response combination module integrates all outputs to produce the final classification (Normal/Abnormal), pathology labels, and referral recommendation.

Module specifications. The UPRETINA system comprises: (1) Quality assessment - classifies image quality; (2) Gradeability - determines if image can be interpreted; (3) Laterality - assigns eye laterality (OD/OS); (4) DR detection; (5) AMD detection; (6) GON detection; (7) ERM detection; (8) Nevus detection; (9) Outlier detector - identifies out-of-distribution cases; (10) Response combination - integrates outputs for final decision. Each pathology detection model outputs a binary classification (positive/negative). Each pathology detection model outputs a binary classification (positive/negative). The system architecture is illustrated in Figure 3.

The total data set consisted of 148,027 images, which were divided into 139,816 images, which were subdivided into images for training and validation, and 8,211 images for testing (Casado-García et al., 2022). The test set only contains images from the CatSalut dataset, while both the training and validation sets contain images from both the CatSalut and Kaggle dataset. The clinical and demographic characteristics of the patients and the images used to create the DR algorithms have been previously published in detail (Casado-García et al., 2022). Additionally, the images data sets of AMD (Zapata et al., 2021) and the other pathologies (Zapata et al., 2017; Zapata et al., 2021) were obtained from the UPRETINA database. The performance of the UPRETINA diagnostic system is summarized in Table 1.

**Table 1:** Performance of the UPRETINA diagnostic system.

Study	Year publication	CNN	Disease	AUC	Type of study
Zapata et al. (2020)	2020	AMDNET	AMD	0.93	Laboratory
Zapata et al. (2020)	2020	RESNET50	GON	0.86	Laboratory
Casado-García et al. (2022)	2022	ResNetRS	DR	0.79	Laboratory
Casado-García et al. (2022)	2022	ResNetRS	DR	0.85	Laboratory
Domínguez et al. (2023)	2023	Inception v4	AMD	0.95	Laboratory
Domínguez et al. (2023)	2023	EfficientNet v2	AMD	0.96	Laboratory
Domínguez et al. (2023)	2023	EfficientNet B3	AMD	0.96	Laboratory
de Vente et al. (2024)	2024	ResNet-RS-50	GON	0.95	Laboratory
Font et al. (2022)	2022	custom neural network architec- ture	AMD	0.98	Clinical validation
Font et al. (2022)	2022	InceptionV3	DR	0.95	Clinical validation
Font et al. (2022)	2022	ResNet50	GON	0.89	Clinical validation
Font et al. (2022)	2022	InceptionV3	Nevus	0.93	Clinical validation

CNN, Convolutional Neural Network; AUC, Area under curve; DR, Diabetic retinopathy; AMD, Age related macular Degeneration; GON, glaucomatous optic neuropathy.

### 2.7 Statistical analysis

A standard statistical analysis was performed using SPSS Statistical Software Version 23.0 (IBM SPSS Statistics for Windows, Armonk, NY: IBM Corp).

The area under the receiver operating curve (AUROC) was used to assess the algorithm performance as compared to the reference pattern. Sensitivity and specificity, with their corresponding 95% confidence intervals (95%CI) were calculated.

Inter- and intra-observer agreement was assessed by using the Kappa correlation coefficient. We additionally assessed the F1 score.

### 2.8 Reporting standards

To ensure the highest level of reporting integrity and transparency, this study has been documented in accordance with the Standard for Reporting of Diagnostic Accuracy Studies (STARD) guidelines (Cohen et al., 2016) and following the Guidelines on Clinical Research Evaluation of Artificial Intelligence in Ophthalmology (Yang et al., 2023).

### 2.9 Computational requirements

Computational requirements. The system processes images in real-time, with mean inference latency compatible with clinical workflow requirements in primary care screening settings. The end-to-end latency per image and peak memory consumption are within typical constraints for deployment on standard clinical workstations. A time comparison for each algorithm of the system is provided in Table 2.

**Table 2:** Computational cost of each model included in the UPRETINA diagnostic system measured in mean (std) inference time in miliseconds using a GPU Nvidia RTX 2080 Ti with 11 GB RAM, number of parameters (in millions) and MACs (in billions).

Model	Mean (std) inference time	Parameters	MACs
DR	319 (6.04)	33.6	23.4
AMD	193 (1.72)	10.6	7.1
GON	906 (10.4)	87.5	82.2
ERM	812 (51.7)	87.5	82.2
Nevus	782 (17.7)	87.5	82.2
Quality	38.3 (3.29)	3.2	0.984
Laterality	9.58 (0.3)	0.8	0.065

## 3 Results

**Figure 4:**
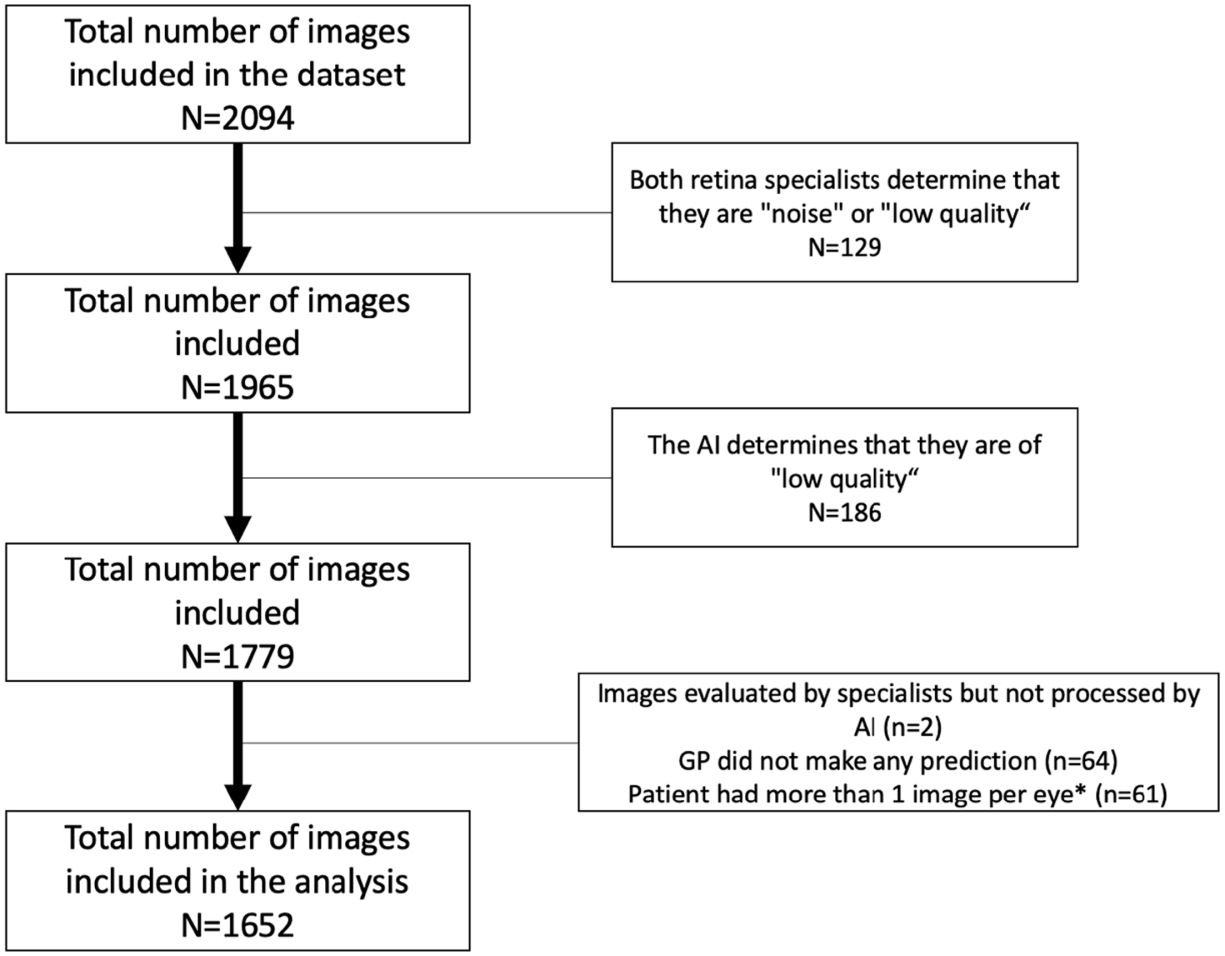
Study flowchart. *The image with the lowest quality was removed of the analysis.

A total of 902 patients, 554 (61.4%) men and 348 (38.6%) women, were evaluated. Of the total of 2094 images included in the dataset, 442 (21.1%) were excluded for different reasons. Therefore, a total of 1,652 images were included in the analysis (Figure 4). The mean age was 65.4.

### 3.1 Ability of the AI algorithm to detect laterality

This study evaluated the capacity of the AI algorithm for detecting the image as right or left eye in the study sample. The sensitivity /specificity for this item was 1.0/1.0, respectively.

### 3.2 Ability of the AI algorithm to assess image gradeability

The study also analyzed the sensitivity/specificity of the AI algorithm to assess the image gradeability (medium or high quality). Of the total images sample, the AI algorithm showed a sensitivity/specificity of 0.9291/0.9053, respectively.

### 3.3 Diagnostic capability

An overview of the sensitivity/specificity values of the AI algorithms and primary-care specialists for detecting different retinal pathologies have been summarized in Table 3.

**Table 3:** Overview of the sensitivity/specificity achieved by the artificial intelligence (AI) algorithm and the primary-care specialist (PCS) as compared to retina specialist ophthalmologists (RSO), for detecting different retinal pathologies in the total images (*n* = 1,652).

	AI algorithm	PCS
Patient referral*		
Sensitivity	0.8988 (0.8788–0.9188)	0.4606 (0.4275–0.4937)
Specificity	0.7099 (0.6798–0.7400)	0.8600 (0.8370–0.8830)
DR		
Sensitivity	0.8684 (0.8521–0.8847)	0.5964 (0.5727–0.6201)
Specificity	0.9564 (0.9466–0.9662)	0.9414 (0.9301–0.9527)
AMD		
Sensitivity	0.9487 (0.9381–0.9593)	0.5128 (0.4887–0.5369)
Specificity	0.9442 (0.9331–0.9553)	0.9237 (0.9109–0.9365)
Nevus		
Sensitivity	0.8965 (0.8818–0.9112)	0.2758 (0.2542–0.2974)
Specificity	0.9796 (0.9728–0.9864)	0.9600 (0.9506–0.9694)
ERM		
Sensitivity	0.8695 (0.8533–0.8857)	0.0860 (0.0725–0.0995)
Specificity	0.9746 (0.9670–0.9822)	0.9631 (0.9540–0.9722)
GON		
Sensitivity	0.8269 (0.8087–0.8451)	0.0570 (0.0458–0.0682)
Specificity	0.9237 (0.9109–0.9365)	0.9593 (0.9498–0.9688)

^*^Decision to refer the patient to the retina specialist.

AI, Artificial intelligence; PCS, Primary-care specialist; DR, Diabetic retinopathy; AMD, Age-related macular degeneration; ERM, Epi-retinal membrane; GON, Glaucoma optic neuropathy.

As compared to retina specialist ophthalmologists, the DR AI algorithm had a sensitivity of 0.8684 (95% CI: 0.8521–0.8847) and specificity of 0.9564 (95% CI: 0.9466–0.9662), while the primary-care specialists had a sensitivity/specificity of 0.5964/0.9414 (95% CI: 0.5727–0.6201/0.9301–0.9527) respectively.

Regarding AMD, the sensitivity/specificity was 0.9487/0.9442 (95% CI, 0.9381–0.9593/0.9331–0.9553) and 0.5128/0.9237 (95% CI, 0.4887–0.5369/0.9109–0.9365) for the AI and primary-care specialist, respectively.

In addition, the AI and primary-care specialist had a sensitivity/specificity for detecting glaucoma optic neuropathy (GON) of 0.8269/0.9237 (95% CI: 0.8087–0.8451/0.9109–0.9365) and 0.0570/0.9593 (95% CI: 0.0458–0.0682/0.9498–0.9688), respectively.

Regarding the ability to detect any pathology, the AI algorithm showed a sensitivity/specificity of 0.8190/0.9797 (95% CI, 0.8004–0.8376/0.9729–0.9865). The sensitivity/specificity of the AI algorithm for detecting vision threatening retinopathy (severe/proliferative DR) was 0.8750/1.0 (95% CI: 0.8591–0.8909/1.0–1.0), respectively. There were only 7 cases in the study, so these results should be interpreted with caution.

Area under the receiver operating curve (AUROC) Degree of agreement, and Accuracy.

The AUROC of the AI algorithm ranged between 0.9122 (GON) and 0.9777 (AMD) (95% CI: 0.8986–0.9258 and 0.9706–0.9848 respectively).

Among all the different pathologies analyzed, the greater Kappa index of both AI and primary-care specialist were, in general terms, those corresponding to the DR. It was probably due to primary-care doctors are trained to assess DR and not as well trained to assess glaucoma or nevus.

Additionally, the degree of accuracy of the AI ranged between 0.9207 (GON) and 0.9782 (Nevus) (95% CI: 0.9077–0.9337 and 0.9712–0.9852 respectively); while in the primary-care specialists ranged from 0.9140 (AMD) and 0.9509 (ERM) (95% CI: 0.9005–0.9275 and 0.9405–0.9613 respectively).

Table 4 summarizes the AUROC, degree of agreement, and accuracy achieved by the artificial intelligence algorithm and the primary-care specialist as compared to retina specialist ophthalmologists.

**Table 4:** Overview of the degree of agreement achieved by the artificial intelligence (AI) algorithm and the primary-care specialist (PCS) as compared to retina specialist ophthalmologists (RSO), for detecting different retinal pathologies in the total images (*n* = 1,652).

	Overall study sample (*n* = 1,652)		
	Retina specialist	AI algorithm	PCS
Patient referral*			
AUROC	NA	NA	NA
Kappa index	0.5478	0.4405	0.3200
Accuracy	NA	0.7485 (0.7276–0.7694)	0.7784 (0.7584–0.7984)
DR			
AUROC	NA	0.9538 (0.9437–0.9639)	NA
Kappa index	0.6825	0.6810	0.4564
Accuracy	NA	0.9503 (0.9398–0.9608)	0.9176 (0.9043–0.9309)
DR Classification			
AUROC	NA	NA	NA
Kappa index	0.6188	0.7126	0.3662
Accuracy	NA	NA	NA
AMD			
AUROC	NA	0.9777 (0.9706–0.9848)	NA
Kappa index	0.5516	0.4250	0.1897
Accuracy	NA	0.9443 (0.9332–0.9554)	0.9140 (0.9005–0.9275)
Nevus			
AUROC	NA	0.9641 (0.9551–0.9731)	NA
Kappa index	0.3995	0.5810	0.1315
Accuracy	NA	0.9782 (0.9712–0.9852)	0.9491 (0.9385–0.9597)
ERM			
AUROC	NA	0.9214 (0.9084–0.9344)	NA
Kappa index	0.3845	0.1402	0.0270
Accuracy	NA	0.9746 (0.9670–0.9822)	0.9509 (0.9405–0.9613)
GON			
AUROC	NA	0.9122 (0.8986–0.9258)	NA
Kappa index	0.2745	0.3659	0.0140
Accuracy	NA	0.9207 (0.9077–0.9337)	0.9309 (0.9187–0.9431)

Kappa in the retinal specialist’s column refers to interobserver concordance, while kappa in the other columns refers to concordance with the retinal specialists.

^*^Decision to refer the patient to the retina specialist.

AI, Artificial intelligence; PCS, Primary-care specialist; NA, Not applicable; ND, Not determined; DR, Diabetic retinopathy; AMD, Age-related macular degeneration; ERM, Epi-retinal membrane; GON, Glaucomatous optic neuropathy.

**Figure 5:**
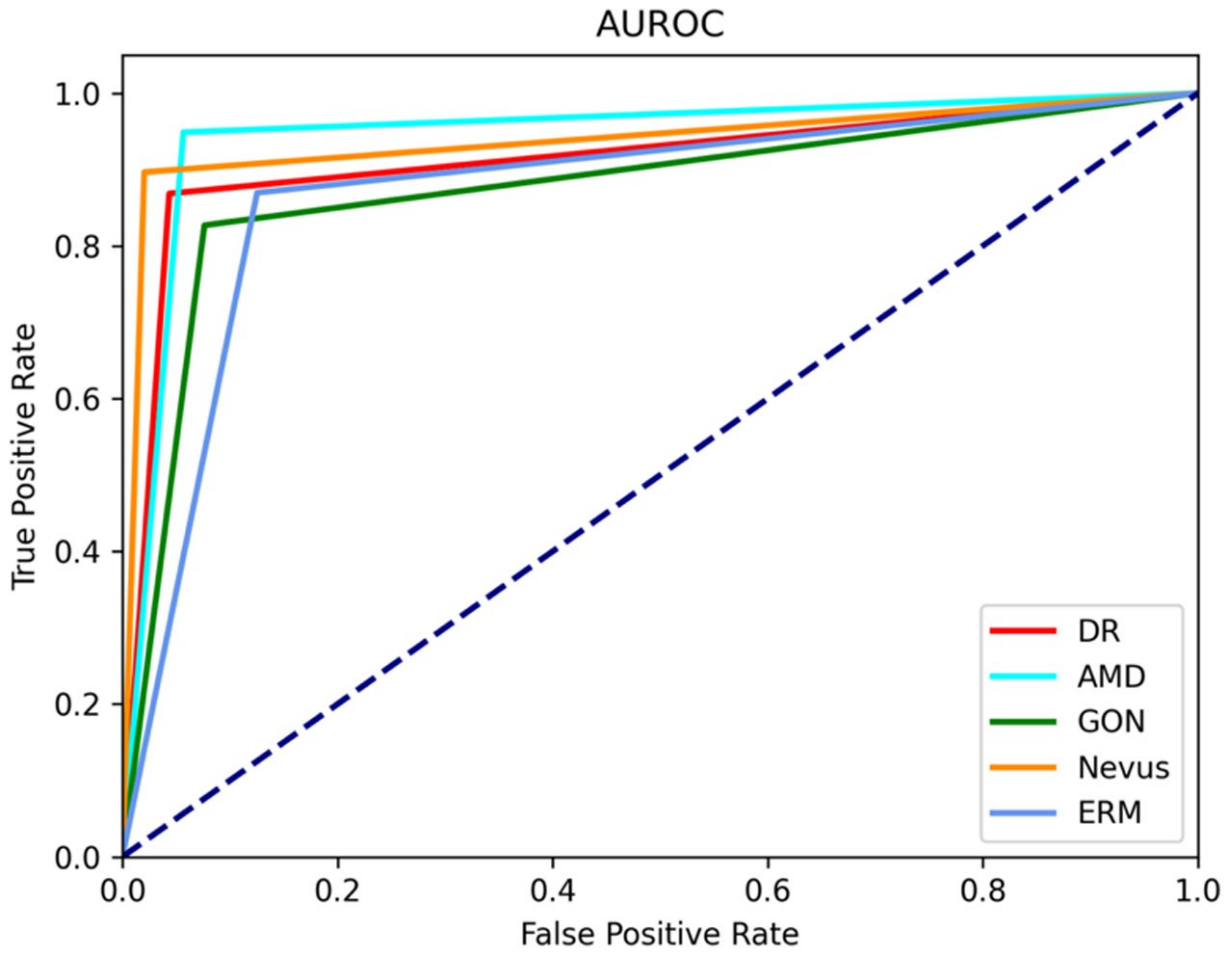
Receiver operating characteristic (ROC) curves for the diagnostic capability of the artificial intelligence (AI) algorithm for the different pathologies. DR AUROC: 0.9538; 95% CI: 0.9437–0.9639; AMD AUROC: 0.9777; 95% CI: 0.9706–0.9848; GON AUROC: 0.9122; 95% CI: 0.8986–0.9258; Nevus AUROC: 0.9641; 95% CI: 0.9551–0.9731; ERM AUROC: 0.9214; 95% CI: 0.9084–0.9344. DR, Diabetic retinopathy; AMD, Age-related macular degeneration; GON, Glaucomatous optic neuropathy; ERM, Epi-retinal membrane; CI, Confidence interval.

Figure 5 shows the Receiver Operating Characteristic (ROC) curves of the artificial intelligence algorithm by taking the optimal point for sensibility and specificity — this value was obtained based on the binary nature of the models.

## 4 Discussion

The current paper aimed to assess the clinical performance of different AI algorithms of the UPRETINA system for diagnosing different center-involved retinal diseases as compared to retina-specialist ophthalmologists.

The results of this study found that this system had a sensitivity/specificity of 0.8684/0.9564 for diagnosing DR; 0.9487/0.9442 for diagnosing AMD; 0.8269/0.9237 for diagnosis GON; 0.8965/0.9796 for diagnosing Nevus; and 0.8695/0.9746 for diagnosing ERM. Additionally, the AUROC of the UPRETRINA system was ≥0.91 for all pathologies studied.

It is important for general practitioners to promptly diagnose and refer sight threatening diseases, including DR, AMD, GON, or ERM to the ophthalmologist for further evaluation, work up, and treatment.

In this study a group of specialists in family medicine were also chosen as comparators. The results of the current study found that, as compared to retina specialist ophthalmologists, the primary-care specialists had a sensitivity/specificity of 0.5964/0.9414 for diagnosing DR; 0.5128/0.9237 for diagnosing AMD; 0.0570/0.9593 for detecting GON; 0.2758/0.9600 for detecting nevus; and 0.0860/0.9631.

These results suggested that an UPRETINA tool can accurately detect different center-involved retinal diseases and GON and prompt referral for ophthalmic evaluation when necessary.

Early diagnosis of central-involved retinal diseases, particularly DR and diabetic macular edema (DME), allows the administration of treatments which significantly reduced vision impairment and, therefore, to preserve patients’ quality of life (QoL) (Garg and Davis, 2009).

Different international recommendations have been developed for detecting diabetes-related eye diseases at early stages, before deterioration of visual function and QoL occur (Wong et al., 2018; Gale et al., 2021). In Spain and other European Union countries, DR screening is performed by fundus photography with a non-mydriatic camera every 12–24 months, depending on whether has been detected (Rodriguez-Poncelas et al., 2015; Lanzetta et al., 2020). In fact, current evidence suggests that performing regular fundus examinations in diabetic patients, using a non-mydriatic camera, is an effective strategy to control and prevent the onset of diabetes-related ophthalmological diseases (Garg and Davis, 2009; Sánchez González et al., 2017; Chan et al., 2015). Furthermore, the examination of the fundus with a non-mydriatic camera, in addition to being a simple and accessible technique, presents great clinical performance due to its high sensitivity/specificity (Piyasena et al., 2019).

Moreover, from a clinical perspective, despite an overall increase in type-2 DM prevalence, not only in Spain (Soriguer et al., 2012), but also worldwide (Ogurtsova et al., 2017; Harding et al., 2019), a decrease in DR prevalence, particularly vision threatening DR (VTDR), has been observed (Harding et al., 2019). This reduction might be explained by the improvements in patients care and the implementation of early-diagnosis programs (Rodriguez-Poncelas et al., 2015).

Nowadays, there has been a growing interest in the development of AI and ML-based systems for screening and diagnosing different ophthalmology diseases, including DR, glaucoma, AMD, as well as for predicting the prognosis of other ophthalmic diseases, such as glaucoma (Somfai et al., 2014; Raman et al., 2019; Venhuizen et al., 2017; Ittoop et al., 2022).

Good proof of this is that the World Health Organization has recently published its first global report on Artificial Intelligence (AI) in health (World Health Organization, 2021). This report concludes that AI can improve the speed and accuracy of diagnosis and screening for diseases, with the subsequent strengthen in health interventions, health research, and drug development, as well as allows patients to greater control of their own health care (World Health Organization, 2021).

Moreover, these strategies need to prove to be equivalent to the diagnostic performance of ophthalmology specialists.

Although 20% of the images were obtained after pupillary dilation, it should be noted that the remaining 80% were performed without pupillary dilation (see Supplementary Table S2), which gives it an important clinical utility to this algorithm. Other AI algorithms required pupillary dilation to obtain good quality images (Rajalakshmi et al., 2018; Paul et al., 2022). For example, the IDx software (IDX, LLC, Coralville, Iowa, USA) was unable to perform approximately 40% of the cases without pupil dilation (Paul et al., 2022).

Current evidence suggests that AI-based devices have a high accuracy with high sensitivity and specificity for diagnosing DR, against various reference standards (Abràmoff et al., 2018; Rajalakshmi et al., 2018; Paul et al., 2022; Gargeya and Leng, 2017; Ting et al., 2017; Verbraak et al., 2019). However, it should be mentioned that these algorithms consider as “healthy” eyes with mild DR; which could have increased their sensitivity/specificity values.

On the contrary, UPRETINA system can identify patients with mild DR. Although the clinical implications of mild forms of DR may be limited, identifying patients early in the process may be helpful for their management.

Additionally, the implementation of AI DR screening tools in primary-care has been associated with a significant improvement of patient adherence to eye-care recommendations and reduced the need to specialist referral (Liu et al., 2021). Moreover, among patients referred to ophthalmologist for clinical evaluation, the rate of adherence was significantly greater in patients who underwent AI screening as compared to the historical adherence rate (55.4% versus 18.7%, *p* < 0.0001) (Liu et al., 2021).

As the population ages, it is crucial to discover cost-effective methods for early diagnosis (World Health Organization, 2025). The cost-effectiveness of population-based screening programs has been demonstrated to be dependent on the frequency of retinal examinations and retinal imaging (Dismuke, 2020). In this regard, the use of AI devices for DR diagnosing has shown to be cost-effective when compared to conventional screening tools (Xie et al., 2020; Gomez Rossi et al., 2022; Huang et al., 2022).

However, the clinical- and cost-effectiveness of the AI tools critically depends on an increase of the referral compliance of patients with suspected DR increases, especially in low- and middle-income countries (Lin et al., 2023).

In addition to DR, it would be extremely important to include other center-involved retinal diseases. UPRETINA evaluates five key ocular pathologies simultaneously. Apart from DR, the algorithms also include AMD, GON, nevus and epiretinal membrane, offering a more versatile approach than systems focused on a single disease. AMD is currently considered as one of the major causes of central vision loss in developed countries, with an estimated global prevalence of 196 million people in 2020 (Wong et al., 2014).

Previous reports have indicated that conducting age-related macular degeneration (AMD) screenings every 5 years in patients aged 40 and above can reduce the incidence of blindness caused by this condition by more than 40% (Tamura et al., 2022).

Although different studies have recently evaluated the use of AI in eyes with AMD, to date, there is still no consensus about its use on fundus photographs, whether associated or not with an OCT scan (Schmidt-Erfurth et al., 2018). Current evidence, indeed, supports the concomitant use of retinographies and OCT scans for improving the sensitivity and reliability of the results (Ipp et al., 2021; Thakoor et al., 2022).

In the current study, the UPRETINA algorithm had a sensitivity/specificity of 0.9487/0.9442 for detecting AMD.

AMD classification in this study was performed according to the International Classification and Grading System, which is the reference standard for fundus photograph-based AMD grading and does not require OCT confirmation. Both human graders and the AI system were evaluated against this same color fundus photography-based standard. Therefore, the absence of OCT did not introduce differential bias between AI and human assessment, nor did it affect the validity of AMD classification within the scope of this screening context.

The current study also investigated the sensitivity, specificity, and the accuracy of UPRETINA system for detecting the presence of ERM (0.8695, 0.9746, and 0.9746, respectively).

Few studies have evaluated the ability of AI to detect ERM (Dong et al., 2022; Gu et al., 2023). Dong et al. (2022) reported a sensitivity/specificity of 97.8%/90.6% and a F1-Score of 0.600 for detecting ERM, while Gu et al. (2023) informed of a sensitivity/specificity of 96%/94% and an accuracy of 0.94 for detecting ERM.

The results of the current study are in line with these studies, although it is difficult to perform direct comparisons since we evaluated different AI algorithms.

Finally, this study also evaluated the UPRETINA system performance for diagnosing GON. Its sensitivity/specificity was 0.8269/0.9237, with an AUROC of 0.9122. These results agree with those published by Ting et al. (2017) and Li et al. (2018).

In screening systems, it is very important that no pathological patient goes without seeing an ophthalmologist. In our study, the negative predictive values are very high (0.99) (Supplementary Table S1). The presence of false positives is common and acceptable in screening systems, which is why other authors have found that the combination of hybrid systems with AI and human reading can be even more cost-effective (Xie et al., 2020).

The algorithm is planned to be integrated into the primary care Electronic Health Records, enabling seamless use by healthcare professionals. Beyond sensitivity, specificity or other metrics, it is necessary to evaluate the utility of these systems in routine clinical practice and how their use integrates both in stand-alone systems and hybrid systems.

This study was conducted within a diabetes screening program in a specific regional population, which likely enriches the cohort for diabetic retinopathy while underrepresenting isolated age-related macular degeneration or glaucomatous optic neuropathy typically found in broader non-diabetic populations. This context may introduce spectrum bias and limits the generalizability of the results beyond a diabetic screening setting.

The low prevalence of several conditions, particularly AMD and GON, in this diabetic screening cohort limits the precision of performance estimates and the strength of conclusions regarding their screening capability. These results should therefore be interpreted as exploratory and context-dependent, rather than definitive evidence of screening performance for these pathologies.

It would be, therefore, necessary to expand the sample of pathological images. In addition, the system of inclusion of the analyzed images represents another limitation of this study. Because the gold standard was evaluation by retina specialists, those images that did not present enough quality for ophthalmologists were discarded; while in real practice the IA would be the responsible of discarding low resolution images; without human intervention. Nevertheless, it should be highlighted that both sensitivity and specificity in this area were above 90%.

One of the main limitations of retina screenings, whether with human reading or AI, is the quality of the images. This is why we believe that the inclusion of real-time quality algorithms can help technicians determine if the images will be adequate or if, on the contrary, the patients need to be dilated or directly referred to an ophthalmologist. Finally, the demographic characteristics of participants were incomplete. Therefore, we were unable to investigate whether there is a relationship between the system’s decisions and some of these characteristics, such as ethnicity.

## 5 Conclusion

Based on the results of this study, UPRETINA system algorithms were capable of automatically and accurately classifying all the screening retinographies, which obviously have a significant impact on reducing workload. Additionally, it has been shown to be capable of distinguishing pathological images from non-pathological ones, leads us to a scenario of more efficient optimization of resources, in which retina specialists will be focus on assessing only pathological images. This would also reduce the primary care burden.

The main limitations when implementing an AI tool in clinical practice are, on the one hand, the lack of integration and homogenization of computer systems and, on the other, the great segmentation and fragmentation of Health Services. In order to solve or reduce the impact of these issues it should be necessary to improve the access, training, and functionalities of retinography devices, as well as to develop a patient journey that takes into consideration the different center-involved retinal diseases.

AI has not been developed for replacing practitioners but rather for helping primary-care and ophthalmology specialists to optimize their time, which allows them to focus on strategies that add high-quality value to healthcare.

The incorporation of a greater spectrum of data, always maintaining and guaranteeing patient confidentiality, will help to establish patient-tailored health care.

## Data Availability

The original contributions presented in the study are included in the article/Supplementary material, further inquiries can be directed to the corresponding author.
