# Supplementary material for: Clinical validation of artificial intelligence algorithms for the detection of different central-involved retinal pathologies and glaucoma from non-mydriatic images
Source: Front Artif Intell. 2026 Mar 10;9:1754682. doi: 10.3389/frai.2026.1754682 (PMC13008691; doi:10.3389/frai.2026.1754682)
Supplement: Supplementary file 1 [file Data_Sheet_1.docx]

Supplementary Material

**Figures**

**Figure S1. Confusion matrices for the diagnostic capability of the artificial intelligence (AI) algorithm for the different pathologies.**


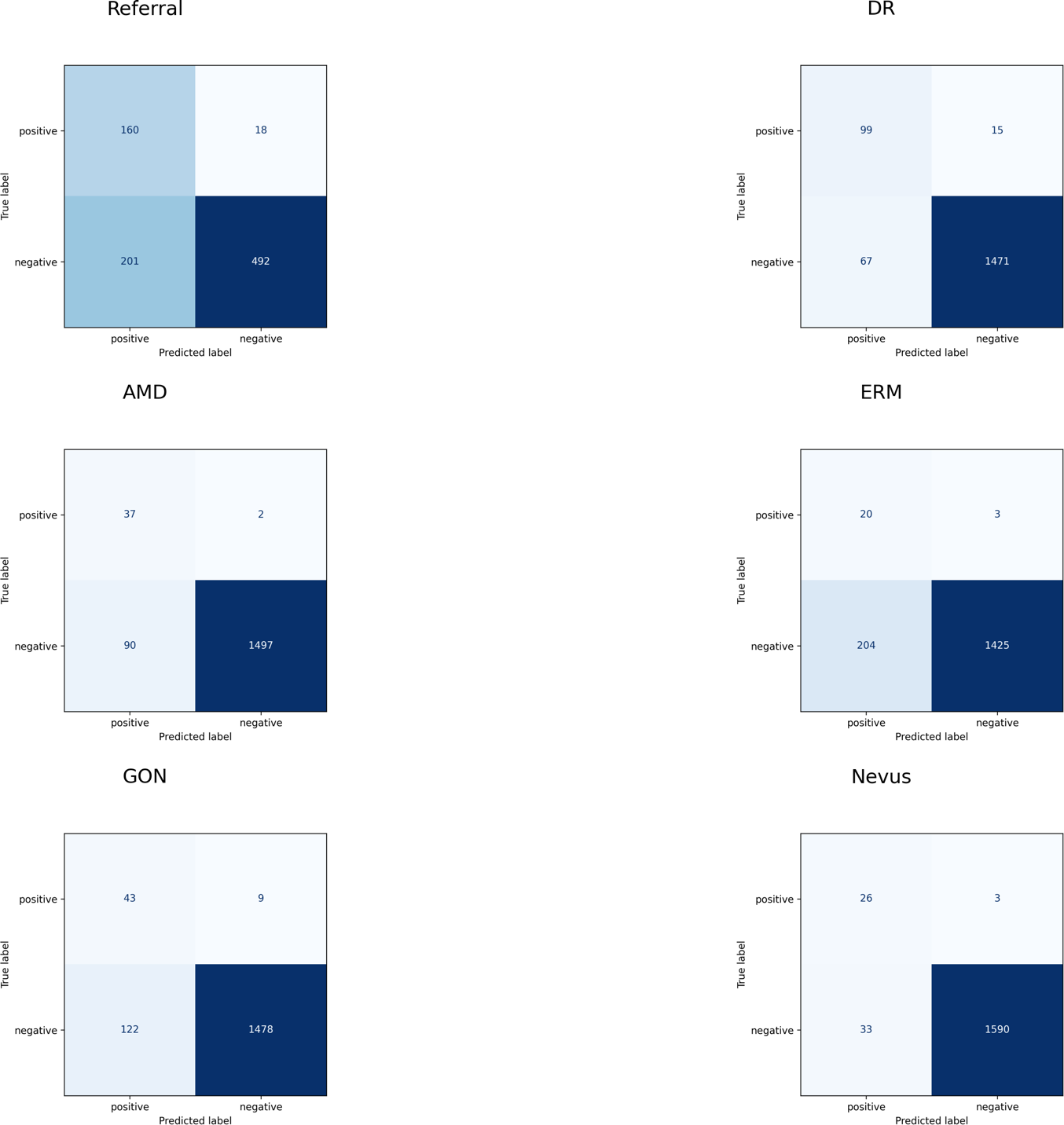


DR: Diabetic retinopathy; AMD: Age-related macular degeneration; GON: Glaucomatous optic neuropathy; ERM: Epi-retinal membrane.

**Tables**

**Table S1. Overview of the positive and negative predictive values of the algorithms**

|  | PPV | NPV |
| --- | --- | --- |
| DR | 0.5963 (0.5727,0.6200) | 0.9899 (0.9850,0.9947) |
| AMD | 0.2913 (0.2694,0.3132) | 0.9986 (0.9969,1.0000) |
| GON | 0.2606 (0.2394,0.2817) | 0.9939 (0.9902,0.9976) |
| Nevus | 0.4406 (0.4167,0.4646) | 0.9981 (0.9960,1.0000) |
| ERM | 0.0892 (0.0755,0.1030) | 0.9978 (0.9956,1.0001) |

PPV: Positive predictive value; NPV: Negative predictive value; DR: Diabetic retinopathy; AMD: Age-related macular degeneration; GON: Glaucomatous optic neuropathy; ERM; Epi-retinal membrane.

**Table S2. Overview of the sensitivity/specificity achieved by the artificial intelligence (AI) algorithm, for detecting different retinal pathologies in the images obtained after pupillary dilation (n=281) and those performed without pupillary dilation (n=1371).**

| Pathology | Sensisitvity | | | | Specificity | | | |
| --- | --- | --- | --- | --- | --- | --- | --- | --- |
|  | With PD | | Without PD | | With PD | | Without PD | |
|  | Mean | 95% CI | Mean | 95% CI | Mean | 95% CI | Mean | 95% CI |
| DR | 0.85 | 0.81-0.89 | 0.88 | 0.95-0.97 | 0.95 | 0.92-0.97 | 0.96 | 0.95-0.97 |
| AMD | 1.00 | 1.00-1.00 | 0.94 | 0.92-0.95 | 0.87 | 0.83-0.91 | 0.96 | 0.95-0.97 |
| GON | 0.90 | 0.87-0.94 | 0.81 | 0.79-0.83 | 0.92 | 0.89-0.95 | 0.92 | 0.91-0.94 |
| Nevus | 0.89 | 0.85-0.93 | 0.90 | 0.88-0.92 | 0.96 | 0.93-0.98 | 0.98 | 0.98-0.99 |
| ERM | 0.00 | 0.00-0.00 | 0.91 | 0.89-0.92 | 0.88 | 0.84-0.92 | 0.87 | 0.86-0.89 |

PD: Pupillary dilation; DR: Diabetic retinopathy: AMD: Age-related macular degeneration; GON: Glaucoma optic neuropathy; ERM: Epi-retinal membrane.
